# East Meets West: A Whole-Person Approach in Integrated Care

**DOI:** 10.5334/ijic.9003

**Published:** 2025-04-21

**Authors:** Minmin Luo, Kexing Liu, Shanshan Hong

**Affiliations:** 1 Department of Sociology and Social Work, College of State Governance, Southwest University, Chongqing, China; 2 Charge nurse, Stomatological Hospital of Chongqing Medical University, Chongqing, China; 3 Department of Sociology, Jimei University, Fujian, China

**Keywords:** whole-person approach, integrated care, culture-specific, Western medicine, Traditional Chinese Medicine, scoping review

## Abstract

**Introduction:**

Whole-person approach represents a fundamental tenet of integrated care globally. However, there remains a lack of consensus regarding its precise definition and an inclination towards superficial and formal implementation. This study aims to compare the similarities and differences between Western medicine and Traditional Chinese Medicine (TCM) perspectives on the whole-person approach, and their potential implications for integrated care.

**Methods:**

We performed a scoping review search of original articles with a sufficient definition of a whole-person approach published in English (Wed of Science) and Chinese (China National Knowledge Infrastructure) between January 2010 and July 2024. A total of 127 articles deemed relevant to this overview were synthesized using a thematic analysis.

**Results and Discussion:**

The study reveals that both Western medicine and TCM adopt a whole-person approach in integrated care, characterized by multidimensionality, dynamism, capability, and collaboration. However, the study also highlights that the goals and focuses of the whole-person approach in Western medicine and TCM differ. The research further discusses the importance of developing a genuine integration of the best ideas from both Western medicine and TCM to achieve a comprehensive and effective whole-person approach to integrated care.

**Conclusions:**

This study highlights the culture-specific perspectives in the whole-person approach to integrated care, and also underscores the necessity of integrating the strengths of this approach derived from diverse cultural contexts.

## Introduction

There is an unfortunate tradition of considering the mind and body as separate and distinct entities, which has led to disease-centered treatment approaches for individuals and families [1]. Until the late 1970s, in response to the rapid growth in the number of chronic patients, escalating healthcare costs, expanding drug side effects, and increasing doubts about the efficacy of biomedical treatments in certain cases, healthcare practitioners, such as clinicians, nurses and social workers, began adopting a more integrated view in understanding and managing psychological and physical health needs [2]. This shift was further influenced by the holistic health movement and the ecological perspective that gained prominence in the 1980s [3]. Such integrated approaches, exemplified by the biopsychosocial model, are characterized by treating the whole person, which requires not only understanding a person’s biological aspects but also recognizing their health as encompassing physical, emotional, mental, social, and environmental factors [4,5]. The whole-person approach to integrated care holds significant potential in the 21^th^ century global healthcare systems to improve the cost-efficiency and quality of care for a range of multi-dimensional issues [6]. These issues may include hospice care [7], mental health services [8], post-disaster trauma care [9], services for cancer patients [10], and integrated care for older adults [11], among others.

A whole-person approach is increasingly being emphasized in integrated care across the healthcare systems and policy documents of many countries, including China. Since 2016, the Chinese government has announced the “Outline of the Healthy China 2030 Plan” and the “Healthy China Action Plan (2019–2030)”. These documents discuss applying a whole-person approach to integrated care in health promotion, rather than focusing solely on disease treatment. However, a gap still exists between discourse and practice. Despite efforts by some authors to operationalize the concept, which have led to broader definitions and descriptions encompassing multiple dimensions, a universally accepted definition remains elusive [12]. Furthermore, some researchers have argued that the dichotomy between disease/body and person is so deeply ingrained in Western biomedical thinking that it has become an “instinctive ontology”, not only for biomedical theorists but also for practitioners and patients [13]. Health achievement is often equated with identifying and removing the disease. Some authors have criticized the whole-person approach as merely a “buzz-phrase” without substantial practical application [14]. It is necessary to reintroduce more holistic understandings of organisms, persons, and communities to replace this instinctive ontology. In particular, there has been growing interest in recent years in integrating and synthesizing Western medical and TCM within a whole-person approach to integrated care [15]. This may offer a better option for both patients and global healthcare systems to addressing contemporary health challenges. For example, the WHO Expert Meeting recommended that member states consider the potential use of TCM for managing COVID-19 within their healthcare systems and regulatory frameworks [16].

Rooted in ancient Chinese philosophy, particularly Daoism and Buddhistic, TCM emphasizes the interconnectedness of body, mind, and spirit, recognizing their ongoing interaction with society and nature to foster harmonious coexistence [17]. Its goal is to provide personalized care that addresses the whole person, thereby stimulating the body’s natural healing capabilities and promoting health even in the presence of disease [18]. TCM is not only an ancient therapeutic modality but also a continuous source of contemporary knowledge, contributing to significant breakthroughs in medicine, such as the Nobel Prize-winning discovery of artemisinin by Youyou Tu [19]. The Chinese government has established an integrated medical model that combines TCM and Western medicine [20]. TCM continues to exert a significant influence on the health perspectives and lifestyles of the Chinese population.

To address these issues, this study employs a scoping review to: 1) examine the evolution of the whole-person approach to integrated care in Western medicine; 2) reflect on the whole-person approach in integrated care from the standpoint of TCM; and 3) compare the similarities, differences, and complementarity between Western medicine and TCM perspectives on the whole-person approach, along with their implications for healthcare staff in understanding patients within their life contexts and achieving optimal health outcomes in integrated care. Notably, our main purpose is to highlight the culture-specific views and methods regarding the whole-person approach, rather than emphasize the epistemological positions of TCM perspectives, or deny that Western medicine perspectives on the whole-person approach provide an appropriate methodology for integrated care.

## Methods

We conducted a scoping review of the literature regarding the whole-person approach to integrated care, concentrating on studies published between January 2010 and July 2024 (including accepted manuscripts but excluding preprints). This study employs a scoping review rather than a systematic review, as the primary objective of a systematic review is to evaluate a theory by providing an unbiased synthesis of the collected evidence to inform policy or practice. In contrast, scoping reviews are exploratory in nature and encompass evidence characterized by diverse research methodologies and content [21]. Their aim is to map key concepts and identify research gaps within a clearly defined field of study. Given that this study was exploratory and included literature spanning various levels of the evidence hierarchy across a wide array of research topics, conducting a scoping review was deemed more appropriate.

Following Arksey and O’malley’s five stages of scoping review, which included: 1) identifying the research question, 2) identifying relevant studies, 3) study selection, 4) charting the data, and 5) collating, summarizing and reporting results [22]. We conducted targeted searches based on our review question within the China National Knowledge Infrastructure (CNKI; http://www.cnki.net/), the most comprehensive repository of China-based information resources, and the Web of Science (http://www.isiknowledge.com), the world’s largest scientific citation index. The search string incorporated terms such as “whole-person approach,” “holistic care,” “patient-centered,” and “person-centered,” combined with the term “integrated care” using the operator “AND.” To narrow our search, given that there were over 10,000 results, citations were required to include at least one of the following keywords: “concept,” “definition,” “content,” “element,” “meaning,” “critical reflection,” “philosophical,” or “theory.” Ethical approval was waived by the authors’ review board since this study utilized secondary data rather than relying on informants’ personal experiences.

### Data collection

**Figure 1:**
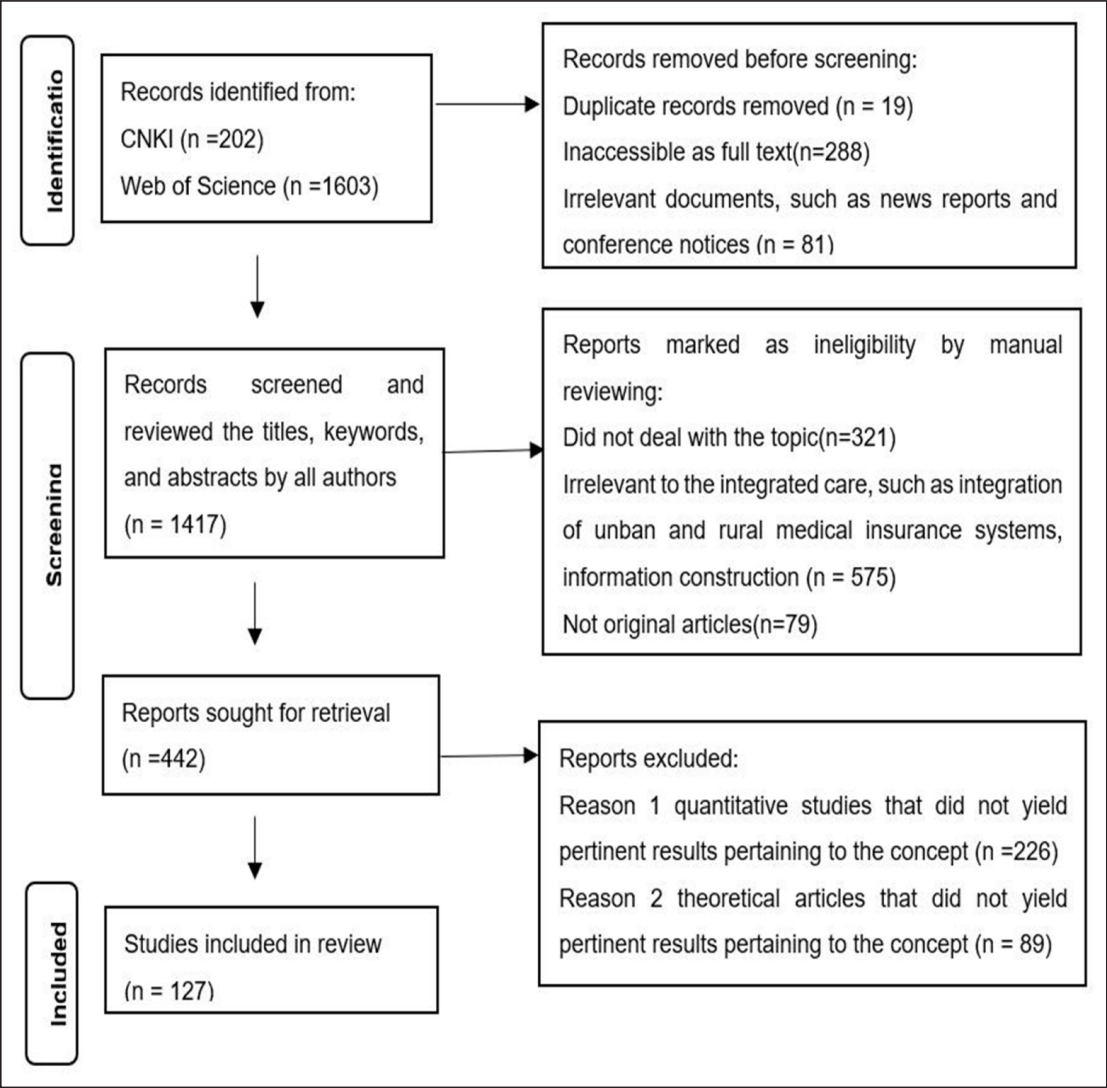
PRISMA diagram.

A total of 1,805 articles were identified through database searches, of which 388 were deemed duplicates or irrelevant. The remaining 1,417 articles were independently screened by the authors based on exclusion criteria (e.g., not original articles). Of these, 442 articles were further reviewed through a comprehensive evaluation of titles, keywords, and abstracts, with full-text reviews conducted as necessary. Quantitative studies and theoretical articles that did not provide relevant insights into the concept were excluded. Any disagreements were resolved through discussion to ensure consensus among the authors. Ultimately, the selection process yielded 127 articles for analysis: 38 Chinese-language articles and 89 English-language articles. The PRISMA diagram is presented in Figure 1.

### Data analysis

The study employed thematic analysis, as outlined by Braun and Clarke (2006) [23], to examine the collected articles’ definitions of the whole-person approach to integrated care and other dimensions describing the concept. The following phases were undertaken: 1) all selected articles were read by all authors and imported into NVivo 12, with initial codes generated; 2) authors read the initial codes to identify potential themes and sub-themes relating to TCM and Western medical perspectives on the whole-person approach to integrated care. Codes with similar content were grouped together. 3) Each theme and sub-theme underwent further scrutiny to ensure accurate reflection of associated codes. The first and second authors conducted repeated reviews and updates to the list of themes and sub-themes through close reading of the articles. Disagreements between reviewers were resolved through discussion or, if necessary, by consulting the third author. 4) The data-driven narrative was produced within and across themes. Table 1 shows examples from some of the articles of codes and subthemes on the theme of multidimensionality.

**Table 1:** Examples of codes and sub-themes on the theme of multidimensionality.


ARTICLE	CODE	SUB-THEME	THEME

Ishikawa et al., 2013, patient-centered care.	the need for obtaining the patient perspective	Combine illness and experience	Multidimensionality

Hudon et al. 2012, patient-centered care.	patients experience illness differently		

Holmstrom & Roing, 2010, patient-centered care.	The uniqueness of eachpatient		

Rosie, 2010, what does ‘patient-centered’ mean	Understanding the whole person (physical, personal, lifestyle and social context, etc.)	Biopsychosocial model	Multidimensionality

Morgan & Yoder, 2012, A concept analysis of person-centered	Beyond inter-personal support (Families, organizations, professions, communities)		

Entwistle & Watt, 2013, Treating Patients as persons	Patients as persons		

Sidani & Fox, 2014, interprofessional care	Care encompasses all domains of health (bio-physical, cognitive, emotional, behavioral, social and spiritual)	Spirituality	Multidimensionality

Tong & Xu, 2018, Spirituality and the importance of a mind–body–spirit approach	The practice of spirituality is multidimensional and multilevel		


We focused on definitions of the whole-person approach to integrated care rather than study results. Therefore, we included a broad range of article types. Given the heterogeneity of the studies, a uniform quality appraisal was less relevant, as the concept’s definition did not depend on study quality. Consequently, we only required that the articles be peer-reviewed and indexed in CNKI and Web of Science.

## Results

The Braun and Clarke approach to thematic analysis identified Western medical and TCM perspectives on the whole-person approach, which were found to encompass four themes: 1) Multidimensionality, 2) Dynamism, 3) Capability, 4) Collaboration. Despite the similarities, there are two notable differences between Western medical and TCM perspectives on the whole-person approach to integrated care. These differences pertain to the specific goals and foci of each perspective in integrated care practice.

### Theme 1: Multidimensionality

The theme of multidimensionality indicates that the whole-person approach encompasses multiple dimensions. These dimensions vary among authors and include the following sub-themes: 1) Combine illness and experience, 2) Biopsychosocial model, 3) Spirituality (see Table 1).

**Sub-theme 1:** Combine illness and experience

In 1969, Enid Balint emphasized that the treatment of diseases cannot solely rely on medication but necessitates incorporating patients’ comprehension of diseases, emotional experiences, and effective communication with others—particularly doctors and nurses [24]. Health communication between patients and caregivers has been described as a means to achieve a broader view of health that incorporates patients’ emotions, meaning, and significance regarding their ailments. Consequently, Western integrated healthcare institutions have adopted a patient-centered approach that emphasizes integrating patients’ experiences [25]. For example, Jayadevappa and Chhatre stated that “physicians must spend the necessary time to listen to and understand the patient needs and preferences” [26]. This shift transforms patients from passive recipients of treatment into active participants in their care [27].

Likewise, TCM consistently upholds a holistic and dynamic approach characterized by “holistic thinking and universal connection” in understanding individual health and well-being [28]. The seven emotions theory posits a direct correlation between emotions such as happiness, sadness, and worry and the organs responsible for their regulation, namely the heart, liver, and lung, respectively. A malalignment of the seven emotions is reflected in a dysfunction of the organs of the body. For example, Depression, as defined in Western medical literature, is characterized by a state of low mood. In contrast, within the context of TCM, it is translated as “抑郁” (yì yù), where the character “抑” conveys a sense of blockage. This term suggests that the flow of seven emotional states within the body becomes obstructed, thereby hindering the proper circulation of “气” (Qi, vital energy) [29]. The practice of TCM involves a holistic approach to both psychological and bodily functions [30].

**Sub-theme 2:** Biopsychosocial model

This dimension has been described in various ways, encompassing the physical, mental, and social aspects of patients’ lives, and is formulated as the “biopsychosocial” model [31]. This model asserts that the whole-person approach to integrated care should extend beyond the interpersonal level between caregivers and patients to include community and organizational levels [32]. Care that focuses solely on biological illness without considering the psychological or social factors hampers healing and contributes to poor outcomes [33]. The biopsychosocial model addresses all aspects of social determinants of health and offers a comprehensive cycle of health promotion and preventive services for vulnerable groups [34]. Authors also propose methods to implement this model, including moving beyond the medical-model bias, treating patients with dignity, addressing patients’ complex health and social needs, and being knowledgeable about local referral resources [35,36].

Through Daoism’s yin-yang theory, which reflects the intertwined duality of all things in nature, TCM also emphasizes the harmonious relationship between humans and their environment [37]. Consequently, TCM adopts a holistic perspective that places equal emphasis on both the subjective experience of the patient and the objective environment in which they exist [38].

**Sub-theme 3:** Spirituality

In the 21^th^ century, researchers in Western societies have increasingly acknowledged spiritual health as an integral component of overall well-being, encompassing individuals’ pursuit of meaning, value, and ultimate reality [39]. In this context, the concept of a whole-person approach integrates the spiritual dimension with the physical, psychological, and social dimensions of integrated care services. The dimension was descried as “Holistic care refers to all domains of health (i.e. bio-physical, cognitive, emotional, behavioral, social and spiritual)” [40], and focuses on the connections between different service stages and services. Understanding of diverse cultural backgrounds significantly influence patients’ perceptions of pain, illness, and treatment modalities [41] could also be included in this dimension.

Under the influence of Taoism and Chinese Buddhism, TCM also incorporates the spirituality, emphasizing the harmonization of the body, mind, and spirit [42]. The practice of spirituality is multidimensional and multilevel, encompassing practices such as mindfulness, Taiji, and Qigong. For instance, mindfulness practices facilitate shifts from psychological and social considerations to deeper cultural understandings, enabling individuals to become more attuned to the interplay between internal and external factors within their current context [43]. On this basis, they can discover more effective strategies for addressing health-related challenges [44]. Moreover, the attitude of acceptance and “letting go” of fixations forms the foundation of non-attachment and non-aversion, which is beneficial for achieving peace of mind and mental health [45].

### Theme 2: Dynamism

Due to the gradual and sudden nature of health problems and changes in individuals’ medical, functional, psychological, or social status, as well as their evolving health and life goals [46], the Western whole-person approach to integrated care has been described as a dynamic, interactive, and interpersonal process. This process flexibly adjusts healthcare services in response to changes in patients’ internal health needs and external environmental resources [47]. The Western whole-person approach to integrated care is characterized by the timely, connected and coherent integration across the health system, medical and supportive services [48].

Based on the theories of Yin-Yang and Wu-Xing, TCM interprets diseases as manifestations of imbalances between Yin and Yang. The goal of treatment is “restoration of normal balance and flow in the body, strengthening and enhancing the body’s endogenous resistance to disease and individualization of therapy” [49]. It is believed that the human body has an innate self-healing function that enables it to fight against bacteria [50]. As a result, diagnosis and treatment are continually evolving. For instance, herbal formulas can be modified on a week-by-week basis to adapt to the changing patterns of a particular patient [51]. The whole-person approach to integrated care in TCM also emphasizes the concept of “preventive treatment of disease”, which encompasses both “prevention before diseases” and “preventing disease from exacerbating” [52].

### Theme 3: Capability

The western whole-person approach to integrated care advocates for competency-based services that prioritize the good health of the individuals. On one hand, it emphasizes empowering individuals through measures such as “respecting patients’ choice” [39, fox, 138], providing emotional support, enhancing their capacity for self-determination, developing methods to assist patients in all stages of decision-making, and helping them manage and cope with their health issues [53]. On the other hand, it focuses on strengthening the interpersonal support network surrounding the individual, including caregivers and family members, by leveraging family resources to enable them to support the individual in addressing health challenges while simultaneously reinforcing social support among these networks [54,55].

TCM is inherently capability-centered, focusing on holistic diagnosis and personalized treatment. The holistic diagnostic approach employs “detailed history taking, close observation, tongue and pulse diagnosis and palpation” [42]. It offers a comprehensive and in-depth understanding of “the human body as well as its energetic interaction with the social and natural environment” [49]. It emphasizes assisting individuals in gaining deeper self-awareness by analyzing their needs, capabilities, characteristics, and circumstances to better identify health problems from a systemic perspective [56]. Furthermore, TCM underscores the importance of spiritual and emotional regulation to enhance patients’ self-efficacy and quality of life throughout the treatment process [57]. It facilitates reframing one’s understanding of suffering, reorganizing values and life purposes, and establishing positive interpersonal relationships and effective social support [58].

### Theme 4: Collaboration

Collaboration was described as reducing unnecessary usage of healthcare resources, thereby making healthcare more integrated and accessible as well as more cost-effective globally [59,60]. Collaboration in integrated care has three dimensions. First, interprofessional teamwork within the healthcare systems, which based on a set of shared competencies, roles, information-sharing, responsibilities and accountability [61]. This involves various professionals, including doctors, nurses, counsellors, social workers, rehabilitators and others. Second, inter-sectoral collaboration between the healthcare system and various government sectors, such as agriculture, food production, industry, education, housing, employment, and public health services [62]. Third, collaboration between professionals and non-professionals (e.g. patients, families, neighborhoods) extends services beyond hospital settings to encompass daily life contexts outside medical facilities [63]. This fosters greater equality in the professional-patient relationship [64]. Castro et.al considered “mutually beneficial partnerships” to be the foundation of a whole-person approach to integrated care [65].

TCM also emphasizes collaboration as a core component of the whole-person approach to integrated care, such as providing extended time for doctor-patient interaction and increased opportunities for patients and families to discuss their priorities and participate in care [42]. Notably, TCM believes that patients are direct participants in regulating their health issues on multiple levels. This includes collaborative efforts between doctors and patients to achieve patients’ “self-regulation,” empowering individuals to align with natural laws through virtuous behavior [66], mental nurturing, physical exercise, balanced nutrition, health education, ways to express their emotions, and healthy lifestyle habits, ultimately aiming to attain a state of “harmony” [67].

### Theme 5: differences between Western and Eastern perspectives on the whole-person approach

The findings demonstrated that, despite similarities, distinct differences exist between Western medicine and TCM perspectives on the whole-person approach in integrated care.

**Sub-theme 1:** Functional life vs. Meaningful life

First, both Western medicine and TCM undoubtedly emphasize holism. However, Western medicine tends to focus on specific diseases, often dissecting patients into manageable components (e.g. organs, tissues, cells, or molecules) for diagnosis and treatment, while TCM takes a broader view of an individual’s overall healthy life, mobilizing the body’s positive factors to treat disease [50]. This difference indicates that the goal of the Western whole-person approach is to achieve a functional life for the individual, whereas the goal of the TCM whole-person approach is to foster a meaningful and harmonious life for the individual [68].

For instance, Western medical perspectives on the whole-person approach in integrated care emphasize symptom-based measures and adaptive functioning. This includes using surgical means to remove tumors, reducing the severity of depression or anxiety, and alleviating patient pain [69]. In this framework, psychosocial circumstances are addressed primarily as secondary considerations, and neither the diagnosis nor the treatment of the disease is likely to differ significantly based on this information. Conversely, while TCM may also focus on diagnosing and treating distinct biomedical diseases, especially in contemporary integrative East-West medicine in China [70]. TCM perspectives on the whole-person approach prioritize the dynamic equilibrium of body and mind, as well as understanding the values that patients and their significant others ascribe to their lives [71].

**Sub-theme 2:** Curing vs. Balancing

It is notable that Western medical’ whole-person approach to integrated care, grounded in the analytical method of “what is” within scientific thesis, aim for an objective understanding while scrupulously avoiding fragmentation. Western medicine has been instrumental in achieving breakthroughs in the diagnosis, treatment, and prevention of specific diseases. Paradoxically, this approach involuntarily reinforces the division among individuals as biological, psychological, social, and spiritual entities, thereby perpetuating the Cartesian mind-body duality that it seeks to transcend through integrated care [72,73]. Western medicine itself remains focused on externally imposed interventions such as blocking, stimulating, removing, or replacing pathological conditions or processes, with healing primarily occurring from external sources [42].

However, in TCM, the cure of diseases is not the primary focus; instead, emphasis is placed on achieving balance within the entire system of mind, body, spirit, and environment. It particularly emphasizes Chinese families play a significant role in influencing individual health behavior, healthcare decision-making, and the development of clients’ integrated care plans in a home and community-based care setting [74]. The whole-person approach integrated care is not merely about making individuals feel cared for by service providers; rather, it aims to facilitate their integration into real life and help them discover practical pathways for personal growth [75].

This perspective, characterized by embracing limitations while being proactive, profoundly influences the daily lives of Chinese individuals, shaping their health-related perceptions and behaviors. In this context, integrated care is seen as integral to individuals’ daily lives, not isolated from their life contexts, healthcare providers adopt a “peer” role, acknowledging patients’ unique health experiences, creating a growth-conducive environment, and assisting in enhancing coping abilities for maintaining good health in everyday life [76]. Correspondingly, within the context of a relationship-oriented, integrative and life-based Chinese medical culture, there is not only a focus on therapeutic modalities such as herbal medicine and acupuncture but also significant attention devoted to maintaining overall wellness through daily health practices. These practices include nutritional therapy, Tai Chi exercises, or counseling and a spiritual reflection on the meaning of life aimed at promoting fitness in everyday life.

## Discussion

The concept of the whole-person approach has gained significant popularity in recent years within the realm of integrated care on a global scale [41]. This is particularly evident in primary care, health promotion, effective management of chronic diseases (e.g. diabetes and hypertension), and pain management [77]. The aim of this study is to critically examine how this approach is understood from both Western medical and TCM perspectives through a scoping review. Our findings are in accord with previous studies indicating that definitions of whole-person approach within integrated care are quite heterogenous and unfocused. The unique cultures and values of various countries significantly influence patients’ perceptions of pain, illness, and their understanding of the whole-person approach to integrated care [78,79,80]. For example, capability is a characteristic of both Western medical and TCM perspectives on the whole-person approach to integrated care; however, there are notable differences. In the Westen medical, the primary objective of the whole-person approach is to enable individuals to function effectively. Consequently, conflicts encountered in life are perceived as deficiencies in personal growth, with an emphasis placed on enhancing individual potential to eliminate or mitigate these conflicts [18]. Conversely, in the TCM, the goal of the whole-person approach is to lead a meaningful life. The conflicts that individuals face are viewed as manifestations of various forces interacting within their lives. Capability thus entails recognizing life’s limitations while fostering one’s own initiative to continually achieve a sense of wholeness in existence [81,82]. It is necessary to examine the ways in which we can systematically combine the differences and overlaps in multicultural perspectives on the whole-person approach to integrated care to address diverse health issues, as recent research has indicated [83].

In contrast to earlier findings, the whole-person approach serves as a framework that integrates biological, psychological, social, and spiritual perspectives for needs analysis [84,85]. It emphasizes clinical integration, professional and organizational integration, as well as system integration [86]. However, our findings highlight the value-driven nature of the whole-person approach to integrated care. The whole-person approach possesses intrinsic value guidance that pertains to varying regional contexts as well as different individuals and groups’ value choices and judgments—interconnected with distinct political, economic, and cultural contexts [87]. For example, despite TCM’s emphasis on collaboration, healthcare institutions at different levels and in various sectors in China often prioritize their own interests, partly due to the compartmentalization of government departments [88]. This results in individuals not experiencing a coordinated, seamless, and lifecycle-provision of integrated care services. Additionally, while Western medicine increasingly recommends certain lifestyle changes, it tends to emphasize more general guidelines for individual lifestyle adjustments based on specific diseases [89].

This study not only highlights the culture-specific perspectives on the whole person approach, but also assists practitioners and researchers in developing a genuine integration of the best ideas from both Western medicine and TCM to achieve comprehensive whole-person care. First, a holographic examination is crucial. This includes both Western medicine’s biomedical diagnosis and TCM’s holistic diagnostic methods. In assessing patients’ health problems and needs, it is essential for health professionals to possess the ability to identify strengths while simultaneously considering patients and their environments as an integrated unit. Through listening, dialogue, interactive engagement, and insightful interpretation of holographic information derived from patients’ life contexts, a genuine integration of body, mind, and environment can be achieved. Second, the primary focus of integrated care is to explore patients’ inherent abilities and resources within their life contexts to restore balance and innate healing capacity. This approach leverages individuals’ daily experiences in their home and community environments to develop coping strategies that are specifically tailored to address their illnesses and other challenges. Simultaneously, health professionals integrate modern biomedical approaches with patients’ perspectives on life, aiming to assist patients in promoting positive changes and achieving meaningful life goals. Third, integrated care involves fostering resilience to maintain harmony amidst dynamic environmental changes, with an emphasis on prevention. This includes promptly identifying and addressing health challenges, mitigating their effects, using medications judiciously when necessary, and maintaining optimal well-being even in the face of illness [90].

The present study has several notable limitations. First, due to resource constraints, we limited our search to CNKI and Web of Science. Additionally, since we included accepted manuscripts, we did not search the grey literature. Consequently, some relevant studies may have been overlooked. Second, this study focused exclusively on TCM within Chinese culture; however, ideologies stemming from the administration of the Chinese government and numerous other philosophies in China may also influence the whole-person approach to integrated care. The examination of Western holistic approaches in this research faces similar limitations. Third, it is important to note that the literature review was conducted within a research context rather than a clinical practice setting. Further systematic research is essential to explore routines, strategies, and metrics for addressing the unique challenges encountered when implementing integrated care across specific cultural contexts.

## Conclusion

The comparison of Western medical and TCM on the whole-person approach to integrated care reveals that this approach is a product of philosophical shifts, transformations in medicine, and changes within society. It advocates for the diversity, complexity, developmental nature, and contextuality inherent in the whole-person approach, emphasizing the importance of defining wholeness through cultural contexts. Moreover, it is crucial to integrate the strengths of the whole-person approach to integrated care derived from diverse cultural contexts. For instance, evidence-based allopathic, disease-directed healthcare, which often emphasizes functional life, holds significant value for individuals and constitutes an essential aspect of a meaningful existence——an insight that TCM has gleaned from Western medical practices. Conversely, integrated care in the West can learn from TCM that one cannot cultivate a greater capacity to cope with pain and suffering without acknowledging life’s losses, recognizing patients’ innate capacity to heal, and understanding our limitations. This insight supports the incorporation of a dynamic and individualized model of care.
